# Supplementary material for: Novel Driver Strength Index highlights important cancer genes in TCGA PanCanAtlas patients
Source: PeerJ. 2022 Aug 11;10:e13860. doi: 10.7717/peerj.13860 (PMC9375969; doi:10.7717/peerj.13860)
Supplement: Supplemental Information 2 [file peerj-10-13860-s002.zip › COHORTS/genes plots/age/2021_8_16_14_9_distribution_cohorts_25-29.pdf]

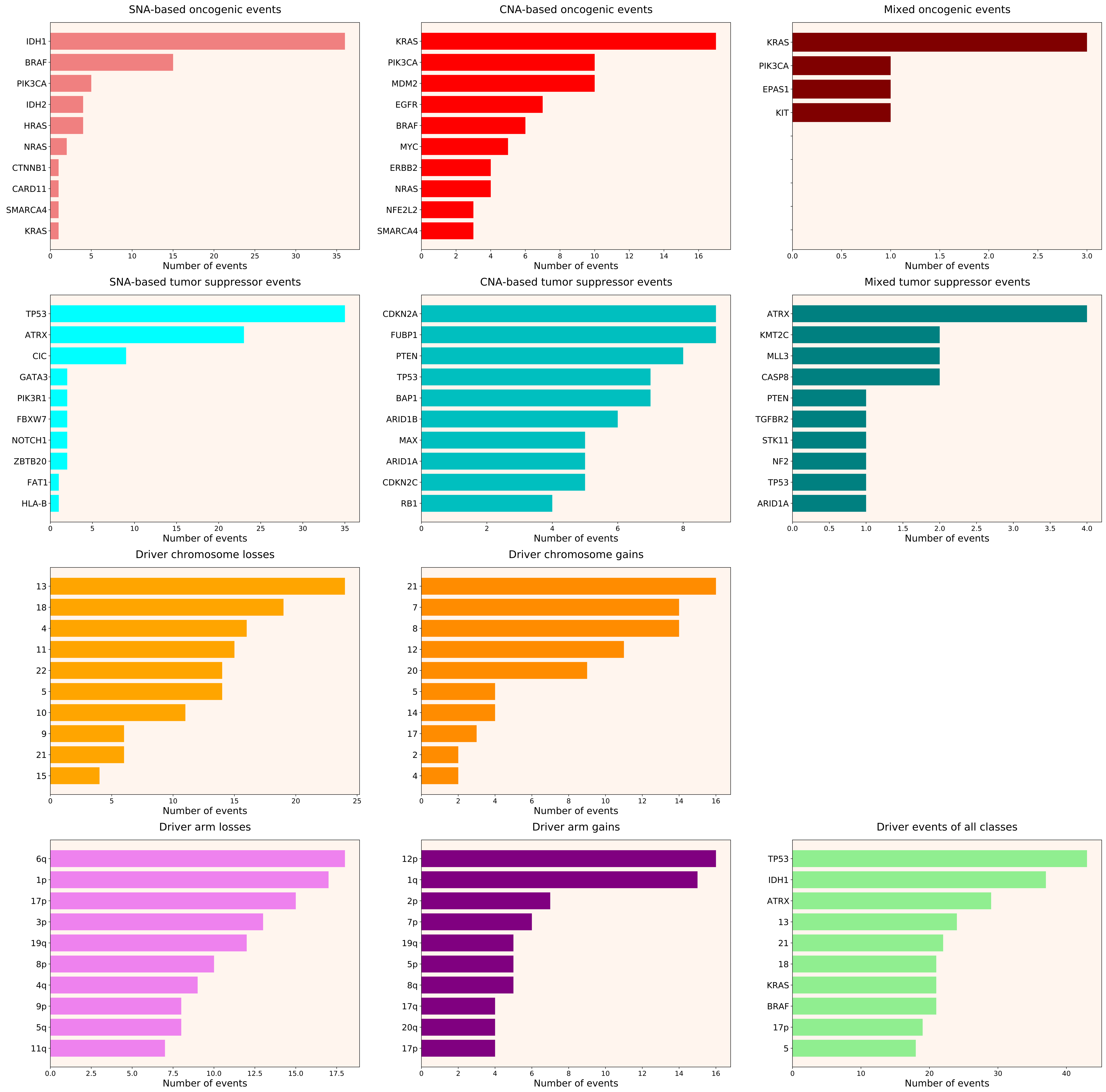
SNA-based oncogenic events
IDH1
BRAF
PIK3CA
IDH2
HRAS
NRAS
CTNNB1
CARD11
SMARCA4
KRAS
Number of events
CNA-based oncogenic events
KRAS
PIK3CA
MDM2
EGFR
BRAF
MYC
ERBB2
NRAS
NFE2L2
SMARCA4
Number of events
Mixed oncogenic events
KRAS
PIK3CA
EPAS1
KIT
Number of events
SNA-based tumor suppressor events
TP53
ATRX
CIC
GATA3
PIK3R1
FBXW7
NOTCH1
ZBTB20
FAT1
HLA-B
Number of events
CNA-based tumor suppressor events
CDKN2A
FUBP1
PTEN
TP53
BAP1
ARID1B
MAX
ARID1A
CDKN2C
RB1
Number of events
Mixed tumor suppressor events
ATRX
KMT2C
MLL3
CASP8
PTEN
TGFBR2
STK11
NF2
TP53
ARID1A
Number of events
Driver chromosome losses
13
18
4
11
22
5
10
9
21
15
Number of events
Driver chromosome gains
21
7
8
12
20
5
14
17
2
4
Number of events
Driver arm losses
6q
1p
17p
3p
19q
8p
4q
9p
5q
11q
Number of events
Driver arm gains
12p
1q
2p
7p
19q
5p
8q
17q
20q
17p
Number of events
Driver events of all classes
TP53
IDH1
ATRX
13
21
18
KRAS
BRAF
17p
5
Number of events
